# Psychometric properties and performance of existing self-efficacy instruments in cancer populations: a systematic review

**DOI:** 10.1186/s12955-018-1066-9

**Published:** 2018-12-27

**Authors:** Fei-Fei Huang, Qing Yang, An-ni Wang, Jing-Ping Zhang

**Affiliations:** 1 School of Nursing, Fujian medical University, No.1 Xueyuan Road, Fuzhou, 350108 Fujian China; 2 Department of Anesthesia, Massachusetts General Hospital, Boston, USA; 3 Xiangya School of Nursing, Central South University, No.172 Tongzipo Road, Changsha, 410013 Hunan China

**Keywords:** Cancer, Instruments, Self-efficacy, Measurement properties, Systematic review

## Abstract

**Background:**

This study aims to provide a systematic compilation of existing measures of self-efficacy developed specifically for use in cancer patients and provide descriptions and comparative evaluations of the characteristics, psychometric properties and performance parameters.

**Method:**

A systematic electronic database search was conducted in PubMed, Ovid (PsyINFO), EBSCO, Elsevier, Scopus to identify self-efficacy assessment tools for cancer patients, between January 1977 to February 2018. The characteristics of target population, instrument, development process and psychometric properties were summarized. All included instruments were subsequently appraised using a psychometric quality assessment tool based on previous publications. Validity of the quality assessment was reviewed and confirmed by five experts.

**Results:**

Fifteen cancer-related self-efficacy instruments were identified. Among them, (40.0%) 6/15 were task-specific, focusing on cancer-related health issues such as fatigue, communication, rehabilitation, exercise, and narcotic pain killer usage. Six instruments were disease-specific for breast cancer, lung cancer, or advanced cancer. Weaknesses of the development processes included the singularity of instrument construction methods, and non-transparent selection of the final items. The main limitation seen in the validation processes was that some important properties of instruments (e.g. test-retest reliability, criterion validity, responsiveness, interpretability, feasibility, and acceptability) were not evaluated.

**Conclusions:**

This review summarizes the limitations and strengths of current self-efficacy instruments for cancer patient. The information reported here can assist clinicians and researchers in the selection of the appropriate instrument. Finally, it points out the need for reporting validation statistics to facilitate the use of these instruments.

**Electronic supplementary material:**

The online version of this article (10.1186/s12955-018-1066-9) contains supplementary material, which is available to authorized users.

## Introduction

Cancer diagnosis, treatment and survivorship challenge the patients’ physical and psychosocial well-being. Cancer patients must manage a number of practical and emotional tasks to cope with the experience over both short and long terms [1]. Self-management empower cancer patients, increasing confidence to manage the disease and treatment, minimizing functional limitations, and enhancing quality of life (QOL) [2]. A critical concept in cancer self-management is self-efficacy, the belief that one can successfully execute behaviors required to produce the expected outcome [3]. For cancer patients, focusing on the positive rather than negative aspects is more beneficial [1, 4]. As a positive psychological resource [1, 5], self-efficacy has received increasing attention for application in life-threatening illness including cancer.

According to Bandura, self-efficacy regulates an individual through cognitive, motivational, affective, and decisional processes to affect one’s motivation to persevere in the face of difficulties [6]. People with high self-efficacy choose to perform more challenging tasks, invest more effort and persist longer [6]. Previous studies showed that among cancer patients, high self-efficacy was associated with increased healthy behaviors (e.g. regular exercise, communication with healthcare providers), greater persistence in achieving the desired psychosocial (e.g. better adjustment, less distress) and physical outcomes (e.g. less pain and fatigue), and higher quality of life [7]. Moreover, self-efficacy is a task-specific construct and must be assessed in-context [6]. For example, self-management in cancer survivorship require coordinating treatments and coping with adverse effects. A particular patient may have high self-efficacy in communicating with providers but low self-efficacy in pain control.

When incorporated into a comprehensive cancer plan of care, instruments that measure self-efficacy inform both researchers and clinicians about patients’ beliefs, capabilities, and motivations [8]. Understanding of self-efficacy can also assist in developing and evaluating programs in health education, self-management intervention, nursing, and psychosocial care [4, 6, 7]. For example, some self-efficacy instruments were used to evaluate the effect of self-efficacy enhancing intervention designed for cancer patients [8, 9].

Toward this goal, a variety of cancer-specific self-efficacy measures have been developed and validated. To ensure robust application of any instrument, a clearly delineated developmental process (e.g. definition of measurement aim, target population, item identification and selection) and critical validation (e.g. characterization of reliability and validity) are required [10–13]. Not knowing whether existing instruments fulfill these quality criteria complicates comparison and selection. To the best of our knowledge, only one systematic review has been published on this subject, which focused exclusively on self-efficacy instruments developed for chronic diseases, such as asthma, arthritis, heart failure, and chronic obstructive pulmonary disease, and did not include cancer [11].

The aim of this study was to provide a systematic compilation of existing measures of self-efficacy developed specifically for use in cancer patients and provide descriptions and comparative evaluations of the characteristics, psychometric properties and performance parameters to help clinicians and researchers select an appropriate instrument.

## Methods

### Inclusion and exclusion criteria

This study was conducted following the guideline of the preferred reporting items for systematic reviews and meta-analysis (PRISMA statement) [14]. Articles were included if they described instruments that aimed at measuring self-efficacy and were developed for used in adult cancer populations. Exclusion criteria were: (1) multidimensional measures comprising a single subscale for the assessment of self-efficacy; (2) approaches using up to several items for self-efficacy without reporting scale development (ad hoc measures); (3) reviews, discussion papers, book chapters, editorials, and reports of purely qualitative approaches to self-efficacy assessment. All studies using a particular instrument were reviewed and evaluated for inclusion. However, if a study only described subsequent applications of the instrument without reporting psychometric parameters, it was excluded from the quality analysis. If an instrument had several versions measuring the same aspects of self-efficacy, only the latest version was included.

### Literature search strategy

A systematic search of the following databases was conducted: PubMed, Ovid (PsyINFO), EBSCO, Elsevier, Scopus. Google Scholar was used as an additional search engine to discover non-duplicate items. Papers published between January 1977 (self-efficacy was first mentioned by Bandura in 1977) to February 2018 assessing the self-efficacy of cancer patients were identified by entering the following keywords or MeSH: “cancer or neoplasm* or oncology* or carcinoma*” AND “self-efficacy or mastery or confiden*” AND “instrument* or scale* or questionnaire or assessment* or measure* or psychometric* or reliab* or valid*”.

No language restriction for the instruments was applied, but only articles published in English were reviewed. The reference lists of all selected studies and reviews were also examined for relevance.

### Study selection

The electronic, multi-database search strategy produced 845 potential studies on self-efficacy in adult cancer populations. Two authors (FFH and QY) independently reviewed the same set of articles and selected the instruments. Discrepancies were resolved by discussion with a third reviewer (JPZ). Authors of the articles were contacted for additional information such as full text when only abstract was found, if needed.

### Data extraction and evaluation

After instrument identification, data were collected in the four main areas: (1) characteristics of the target population (e.g. country, sample size, cancer type, age, gender, education, time since diagnosis and treatment) (Table 1); (2) characteristics of the instrument (e.g. language, frequency of use, administration format, scoring, number of items, domains covered, time needed to complete, reading level, and acceptability) (Table 2); (3) development process (e.g. task focus, construction method, selection and identification of items) (Table 3); and (4) psychometric properties (e.g. reliability, validity, responsiveness, interpretability and floor/ceiling effects) (Table 4 and Additional file 1: Table S1).

**Table 1:** Characteristics of the patient populations used for the initial application of the self-efficacy instruments

Instrument	First author, year [reference number]	Full name	Country	Sample size	Cancer types	Mean age (years)	% female	% high school or above	Year since diagnosis(years)	Treatment(stage& type)
SICPA	Telch, 1986 [16]	The Standford Inventory of Cancer Patient Adjustment	USA	41	Mixed	18~65	NR	NR	NR	NR
SUPPH	Lev et, 1996 [13]	Strategies Used by People to Promote Health	USA	178	Mixed	53.6 ± 13.55	61%	84%	NR	Chemo
SEAC	Hirai, 2001 [26]	The Self-Efficacy scale for Advanced Cancer	Japan	50	Mixed terminal	60.8 ± 9.6	60%	NR	NR	NR
SESCI	Porter et al.,2002^[15]^	Self-Efficacy for Symptom Control Inventory	USA	30	Lung	62.5 ± 10.7	40%	NR	NR	NR
CASE-cancer	Wolf,2005 [20]	The Communication and Attitudinal Self-Efficacy for cancer	USA	136	Mixed	63.3 ± 15.0	15.2%	53.8%	NR	NR
OTSES-CA	Liang,2008 [23]	Opioid-Taking Self-Efficacy Scale-Cancer	China	92	Mixed	56.4 ± 12.2	41%	NR	NR	NR
CBI-B	Heitzmann, 2011 [17]	The brief version of Cancer Behavior Inventory	USA	1304	Mixed	62	71%	41%~ 94%	NR	NR
PSEFSM	Hofffman,2011 [18]	Perceived Self-Efficacy for Fatigue Self-Management instrument	USA	298	Mixed	Age ≥ 21	70%	90%	NR	Chemo +/− XRT
SESSM-B	Lee,2012 [24]	Self-Efficacy Scale for Self-Management of Breast cancer	Korea	303	Breast	47.7 ± 8.7	100%	78.9%	3.4 ± 3.6	53.8% during tx 46.2% post tx 21.5% surgery, 27% XRT 52.8% chemo 3.7% hormone
BCSES	Champion,2013 [25]	Breast Cancer Survivor Self-efficacy Scale	USA	1127	Breast	57.1 ± 11.6	100%	97%	5.9 ± 1.5	Post-chemo
C-SUPPH	Yuan,2014 [14]	Strategies Used by People to Promote Health-Chinese version	China	764	Mixed	54.03 ± 5.13	50.8%	NR	NR	31% surgery + chemo
EBSES	Buchan et al., 2015 [22]	Exercise barriers self-efficacy scale	Australia	101	Mixed	59.6/56.3	92.6%/ 100%	NR	NR	NR
SMSES-BC	Liang, 2015 [8]	Symptom-management self-efficacy scale for breast cancer related to chemotherapy	China	152	Breast	54.3 ± 9.9	100%	75.7%	4.2 ± 5.4	65.2% chemo 37.6% hormone 24.9% target
SMSFS-A	Chan, 2016 [19]	Self-efficacy in managing symptoms Scale-Fatigue Subscale for Patients With Advanced Cancer	Australia	10	Mixed advanced cancer	62.6 ± 9.1	90%	70%	NR	10% XRT 10% chemo+XRT 10% target
SESPRM-LC	Huang et al., 2017 [21]	Self-efficacy scale for rehabilitation management designed specifically for postoperative lung cancer patients	China	448	Lung cancer	58.37 ± 9.9	39.6%	29.7%	NR	Surgery

*NR*: not recorded, *XRT* radiation therapy

**Table 2:** The descriptive characteristics and structure of instruments

Instrument (language if not English)	Mode of administration	Scoring	Number of items	Domains covered		Time needed	Reading level	Acceptability
SICPA^b^	Self	11-point 0 (not at all confident) to 10 (absolutely confident)	38	Six	1) Coping with medical procedures 2) Communication 3) Activity management 4) Personal management 5) Affective management 6) Self-satisfaction	NR	NR	NR
SUPPH^b^	Self	5-point 1 (very little confidence) to 5 (quite a lot of confidence)	29	Four	1) Coping 2) Stress reduction 3)Making decisions 4) Enjoying life.	NR	NR	NR
				Three	1) Positive attitude 2) Stress reduction 3) Making decision.			
				Two	1) Physiological efficacy information 2) Performance efficacy information.			
SEAC^b^ (Japanese)	Self	11-point 0 (not at all confident) to 10(totally confident).	18	Three	1) Symptom coping efficacy 2) ADL efficacy 3) Affect regulation efficacy	NR	NR	NR
SESCI ^b^	Self	10 (not at all certain) to 100 (completely certain)	15	Three	1) Self-efficacy for managing pain 2) Self-efficacy for managing other symptoms 3)Self-efficacy for function.	NR	NR	NR
CASE-cancer ^b^	Interviewer	4-point 1(strongly disagree) to 4(strongly disagree)	12	Three	1) Understand and participate in care 2) Maintain a positive attitude 3) Seek and obtain information	NR	8th grade level or below	NR
OTSES-CA^b^ (Chinese)	Self	11-point 0(not at all confident) to 10(completely confident)	30	Four	1) Pain and the use of analgesics 2)Tailoring of the medication regimen 3)Acquiring help 4) Management of treatment-related concerns.	7.5–20 min (average 11 min)	NR	NR
CBI-B^a^	Self	9-point 1 (not at all confident) to 9 (totally confident)	12	Four	1) Maintaining independence and positive attitude 2) Participating in medical care 3) Coping and stress management 4) Managing affect	NR	NR	NR
PSEFSM ^a^	Self	11-point 0~10(very certain)	6	One	Perceived self-efficacy for fatigue self-management	NR	NR	< 0.005% missing data
SESSM-B^b^ (Korean)	Self	5-point 1 (not at all) to 5(very)	13	Five	1) Coping with psycho-informational demand 2) Maintenance of healthy lifestyle 3) Management of side-effects 4) Therapeutic compliance 5) Sexual life	NR	NR	NR
BCSES ^b^	Self	5-point 1 (strongly disagree) to 5 (strongly agree)	11	One	Self-efficacy of manage symptoms and quality of life problems	NR	NR	NR
C-SUPPH ^a^ (Chinese)	Self	5-point 1 (very little confidence) to 5 (quite a lot of confidence).	28	Three	1) Positive attitude 2) Stress reduction 3) Making decisions.	NR	NR	NR
EBSES ^a^	Self	0% (not at all confident) to 100% (extremely confident), with 10% intervals	5	Two	1) General exercise barriers 2)Lymphedema-specific exercise barriers	NR	NR	NR
SMSES-BC ^a^ (Chinese)	Self	11-point 0(not at all confident) to 10(complete confidence)	27	Three	1)Acquiring problem-solving 2)Managing chemotherapy-related symptoms 3)Managing emotional and interpersonal disturbances	NR	NR	NR
SMSFS-A^a^	Self	11-point: 0 (not confident at all) to 10 (extremely confident)	17	One	Self-efficacy of fatigue self-management behaviors	7.5 min	NR	NR
SESPRM-LC^a^ (Chinese)	Self	5-point 0 (not confident at all) to 5 (completely confident)	27	Six	1) Emotion management self-efficacy 2) Rehabilitation information acquisition and application self-efficacy 3) Coping with treatment adverse effects self-efficacy 4) Symptom self-management self-efficacy 5) Rehabilitation training and skill cultivation self-efficacy 6) Daily life management self-efficacy	NR	NR	NR

Instrument: instrument abbreviation name, asterisks indicate whether a copy of the instrument was provided

^a^ full copy of the instrument

^b^ limited detail on items and scaling information provided, and language. Acceptability reflects the respondents’ willingness to complete the tool and impacts on quality of data, as estimated by percentage of missing data to estimate it

**Table 3:** The development process of included instruments

Instrument	Task focus	Method of construction	Identification of items			
			Expert panel	Patient panel	Data driven	Literature search
SICPA	Various situations or to perform specific behaviors found to be difficult for cancer patients (e.g., asking for help from family members, discussing treatment options with the physician, feeling physically attractive)	CTT	NR	NR	NR	NR
SUPPH	Carrying out self-care strategies	CTT	Y	Y	Y	NR
SEAC	Manage illness behavior of advanced cancer patients	CTT	Y	NR	Y	Y
SESCI	Manage pain, symptoms, and function.	CTT	NR	NR	NR	Y
CASE-cancer	Communicate effectively with healthcare professionals and maintain a positive attitude	CTT & IRT	NR	Y	Y	Y
OTSES-CA	Taking opioids for cancer pain	CTT	Y	Y	Y	Y
CBI-B	Coping with cancer at any point during the course of the disease.	CTT	NR	NR	Y	Y
PSEFSM	Perform fatigue managing behaviors	CTT	Y	NR	NR	Y
SESSM-B	Self-management activities of breast cancer	CTT	Y	Y	Y	Y
BCSES	Manage long-term issues after initial diagnosis and treatment of breast cancer.	CTT	Y	Y	NR	Y
C-SUPPH	Carrying out self-care strategies	CTT	Y	Y	Y	Y
EBSES	Exercise when faced with barriers experienced by individuals with cancer-related lymphedema	CTT	Y	Y	Y	Y
SMSES-BC	Self-management of chemotherapy symptoms of breast cancer	CTT	Y	Y	Y	Y
SMSFS-A	Fatigue management of advanced cancer	CTT	Y	Y	Y	NR
SESPRM-LC	Conducting the rehabilitation activities of postoperative lung cancer	CTT & IRT	Y	Y	Y	Y

**Table 4:** Summary of the quality appraisal of psychometric properties of the included instruments

	Internal consistency reliability	Test-retest reliability	Content validity	Construct validity	Criterion validity	Responsiveness	Interpretability	Floor/Ceiling effects
SICPA	+	+	NR	–	+	+	+	NR
SUPPH	+	+	+	+	NR	+	+	NR
SEAC	+	NR	+	–	NR	NR	+	NR
SESCI	+	NR	+	NR	NR	NR	+	NR
CASE-cancer	+	NR	+	+	NR	NR	NR	NR
OTSES-CA	+	–	+	–	+	NR	+	NR
CBI-B	+	NR	+	+	NR	+	+	NR
PSEFSM	+	NR	+	+	NR	+	+	NR
SESSM-B	–	NR	+	+	+	NR	NR	NR
BCSES	+	NR	+	+	+	NR	NR	NR
C-SUPPH	–	NR	+	+	NR	NR	+	NR
EBSES	+	+	+	–	+	NR	NR	NR
SMSES-BC	–	+	+	+	+	NR	NR	NR
SMSFS-A	NR	+	+	NR	NR	NR	NR	NR
SESPRM-LC	+	+	+	+	+	+	NR	NR

+ positive rating: meeting or exceeding current standards

- negative rating: not meeting current standards

NR not reported

Please refer to Additional file 1: Table S2 for definition of ‘positive’ and ‘negative’ and the individual properties

All included instruments were subsequently appraised using a new quality assessment tool generated by combining and modifying two published tools [11, 12]. The modified tool was also reviewed and confirmed by five psychometric experts. The final form used for evaluation is included in Additional file 1: Table S2.Any discrepancies in the data extraction and evaluation process were resolved by discussion, with additional consultation from two other authors (ZN and JPZ).

## Results

**Fig. 1:**
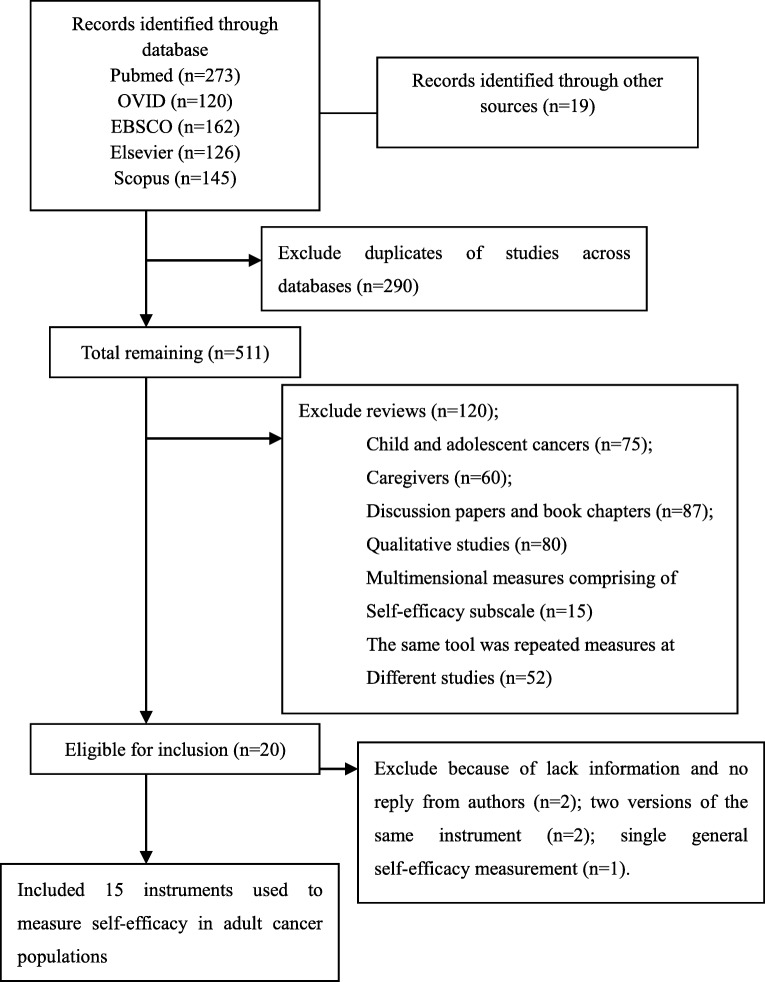
Flow chart of the process of selected studies

As shown in Fig. 1, the initial search strategy identified 845 references after duplicates were removed. Using the inclusion criteria, we retained 15 instruments used to measure self-efficacy in adult cancer populations.

The development of self-efficacy instruments for cancer patients started in 1986, with rising number of new instruments in each of the subsequent decades. Nine (60.0%) instruments measured the self-efficacy of general health strategies in cancer, such as self-care [15, 16], self-management [17], and coping behaviors [18, 19]. Six (40.0%) instruments were task-specific, focusing on fatigue [20, 21], communication [22], rehabilitation [23], exercise [24], and narcotic pain killer usage [25]. Six instruments were disease-specific for breast cancer [10, 26, 27], lung cancer [23] and advanced cancer [21, 28]. We were able to find full administration information on half of the instruments, including list of items, domains, scoring instruments, and format. Nine instruments were developed in English. Four were in Chinese (three were developed in Chinese [10, 23, 25], and one was translated from English to Chinese [16]). One each was in Japanese [28] and Korean [26]. In addition, only one instrument (Strategies Used by People to Promote Health-Chinese version, C-SUPPH [16]) was used in cross-cultural studies to examine the variance across different socio-demographic groups.

### Characteristic of the adult cancer populations studied by the instruments

Seven instruments were initially used in adult cancer populations in the USA, four in China, two in Australia, and one each in Japan and Korea (Table 1). Sample sizes ranged from 10 [21] to 1304 [20]. A variety of cancer types were studied. Four instruments focused exclusively on one cancer: breast [10, 26, 27] or lung [23]. The remaining studies examined mixed cancer groups. Two instruments were used in advanced/terminal cancer populations [21, 28]. Surprisingly, few of the studies included clinical information, such as years since diagnosis, staging and treatments received.

### Instrument characteristics

As shown in Table 2, three instruments were identified as unidimensional (items loading onto one underlying factor), and others multidimensional (items loading onto multiple underlying factors). The dimensions of all instruments were determined and each demonstrated one confirmed factor pattern, except for the SUPPH, which had three structured factor patterns [11, 15]. The number of items included in the instruments ranged widely from five [24] to 38 [18]. Only one instrument [22] was administered by an interviewer. The remainder was self-administered. The majority of instruments used a Likert-type scale.

A total of 54 domains were obtained from all instruments. Nineteen domains focused on self-efficacy in the management of cancer-related physical symptoms, including coping with symptoms or side-effects (15 domains), fatigue management (2 domains) and pain control (2 domains). Eighteen domains were related to psychological management, such as affective management (5 domains), stress reduction (6 domains), positive attitude (5 domains), and problem-solving (2 domains). Eight domains focused on lifestyle, such as activity or exercise (4 domains), sexual life (1 domain) and maintenance of healthy lifestyle (3 domains). Eight domains were about understanding and participating in medical procedures, including communication (1 domain), medical decision-making (5 domains), and information acquisition (2 domains). One domain was general self-efficacy.

Data on the feasibility and burden of administration were scant. While majority of the patients participated in the initial studies had high school education or above, only one instrument, Communication and Attitudinal Self-Efficacy for Cancer (CASE-cancer) [22], specified that it was constructed at the 8th grade the reading level. Two instruments, Opioid-Taking Self-Efficacy Scale-Cancer (OTSES-CA) [25], and Self-Efficacy in Managing Symptoms Scale-Fatigue Subscale for patients with advanced cancer (SMSFS-A) [21], reported the time needed to answer the questions which was less than 20 min. All instruments were administrated by paper-and-pencil. No electronic version was available. One instrument (Perceived Self-Efficacy for Fatigue Self-Management instrument, PSEFSM [20]) reported the minimal missing data as an indication of respondent acceptability.

### Instrument development process

The majority of instruments used the classical test theory (CTT) method for construction, except CASE-cancer and Self-efficacy Scale for Rehabilitation Management for postoperative lung cancer patients (SESPRM-LC) [23], which combined CTT and item response theory (IRT) methods. Expert opinion, patient panel and data from literature were also used to select or screen the items (Table 3). Eleven instruments integrated all three approaches. One instrument (The Standford Inventory of Cancer Patient Adjustment, SICPA) [18] did not report detailed method on identification and selection of items.

### Psychometric properties

The two instruments found to have the most positive ratings in quality assessment were Strategies Used by People to Promote Health (SUPPH) and SESPRM-LC. SUPPH was used to measure the confidence of cancer patients to carryout self-care strategies at any point during the course of the disease. It has been widely used and translated for several languages, including Chinese (SUPPH-C). However, it appeared to not have a stable factor structure,including 2-factor, 3-factor and 4-factor [16]. SESPRM-LC was used to measure the confidence of lung cancer patients to engage in postoperative rehabilitation. As a relatively new instrument, it has robust psychometric properties but needs further testing to establish normal and cut-off values.

The next group of high quality assessment included SICPA, Brief version of Cancer Behavior Inventory (CBI-B) and PSEFSM. Similar to SUPPH, SICPA and CBI-B were used to measure the self-efficacy of general health strategies at any point during the cancer disease trajectory. The SCIPA has some outstanding weaknesses, especially regarding construct validity and content validity. It does not include items that represent management of cognitive tasks or side effects of chemo-radiation, which are critical components of the cancer experience. Compared to the 38-item SCIPA, the 12-item CBI-B presents improved efficiency suitable for screening in clinical settings. However, its reproducibility and concurrent validity wait investigation. PSEFSM was designed specifically for fatigue management and cannot be generalized to other aspects of cancer care.

SMSFS-A had the lowest quality ratings. Due being in the pilot stage of development, the structure and validity of SMSFS-A have not been thoroughly characterized. Although SMSFS-A and PSEFSM both focused on fatigue management, the SMSFS further narrowed down to patients with advanced cancer.

In reliability analysis, 11/15 (73.33%) of instruments had satisfactory internal consistency, with a reported Cronbach’s alpha between 0.75 and 0.95. For the instruments that received negative ratings, two [10, 26] had Cronbach’s alpha > 0.95, indicating redundancy, and one [16] had a subscale Cronbach’s alpha < 0.70. For test-retest reliability, Pearson correlation coefficient was commonly used, with one to two weeks of lapse between two repeated measures. One instrument [25] received negative ratings for Pearson’s *r* < 0.7.

Except for SICPA, all instruments reported content validity by providing feedback from patients, clinicians, experts, or pilot test. Evidence for construct validity was provided for 13 instruments, and four rated negative because of inadequate sample size (< 100). Six instruments used convergent or divergent validity analysis to estimate the degree to which the instrument is correlated with other measures of similar or dissimilar constructs. Ten performed exploratory factor analysis (EFA), and five also used confirmatory factor analysis (CFA) to further confirm construct validity. In studies using EFA, the total variance explained ranged between 43.6% [28] and 81.3% [15]. Four studies examined the construct validity by using both approaches. Criterion validity was reported for seven instruments, and all were positive.

The remaining psychometric properties — responsiveness, floor/ceiling effects and interpretability — were seldom assessed. Only five instruments reported responsiveness. SICPA [18] was sensitive to interventions targeting improvements in self-efficacy. SUPPH [15] detected clinically significant changes over time (at 4 and 8 months). CBI-B [29], PSEFSM [30] and SESPRM-LC [23] detected significant confidence changes pre-and post-interventions. None of the instruments reported cut-offs or normative values, but half provided sample mean scores and standard deviations of at least one patient group to aid in interpretation.

## Discussion

This systematic review examined the psychometric and performance characteristics of 15 existing instruments aimed at measuring self-efficacy in adult cancer populations. An increasing number of self-efficacy instruments have been published in the past three decades. Illustrating Bandura’s self-efficacy theory, most instruments were task- or disease- specific. Our analysis revealed both the strength and limitations of these instruments. While the majority of instruments cover a variety of domains pertinent to cancer self-management and have been tested in clinical situations, their wider applicability is eclipsed by singularities in instrument construction and item selection, and failure to report important psychometric parameters.

Our review confirmed CTT as the most widely used approach for instrument development. Because the respondent characteristic of interest is quantified based on the raw score across all the items in the instrument, score interpretation in CTT is sample specific [31]. To overcome this limitation, IRT was introduced, which is “a diverse family of models designed to represent the relation between an individual’s item response and underlying latent trait” [32]. In IRT, information is obtained at the item level rather than scale level [33]. Only two self-efficacy instrument for cancer patients (CASE-cancer and SESPRM-LC) incorporated IRT method in its construction, which may have helped improve construct validity. We advocate for promoting the application of IRT in future instrument development.

We discovered that a major obstacle in validation is longitudinal assessments. Only a third of the instruments were applied to measure how patients changed over time, with or without self-management intervention. Self-efficacy in cancer likely fluctuates as patients make progress on the path of diagnosis, treatment, and survivorship. Thus monitoring with valid measurements that have high test-retest reliability and sensitive to change becomes paramount. Without adequate data, we cannot discern whether a given instrument can be applied to a proposed intervention. Other areas awaiting improvement in instrument validation include analysis of item performance for refinement purposes, assessment of criterion-based and construct validities in large sample, and identification of cut-off, threshold and normal values to guide interpretation. Most studies did not provide information on clinical practicability of the self-efficacy instruments. Generally, reading level of 8th grade or below and time to completion of no more than 20 min are considered appropriate for cancer patients [34]. In addition, as electronic medical record and research bookkeeping being widely adopted in cancer care [35], investigation into the feasibility of a computer-assisted self-efficacy assessment is highly recommended. These improvements would be necessary for integration of the instruments into daily practice.

Notwithstanding the above considerations, based our review, we recommend SUPPH, SICPA and CBI-B for assessing cancer patients’ confidence in general self-care. For disease-specific instruments, we recommend a breast cancer self-efficacy scale (BCSES) for breast cancer and SESPRM-LC for lung cancer patients.

Our study has several limitations. Only English articles were included,,and the information in dissertations, book chapters, manuals, reviews and other non-peer reviewed or “grey” literature were also not included. Although we paid great attention to the inclusion of instruments that truly measure self-efficacy for cancer patients, we cannot exclude the possibility of having misclassified studies.

### Suggestions for further research

Although self-efficacy is a well-established concept that has been shown to have high explanatory power [6], there is great room for improvement in the assessment. Perhaps more instruments should be developed for specific cancer types challenging the patients with different sets of self-management requirements related to symptoms and treatments. New instruments should take into account the domains summarized in this study: self-efficacy in the management of physical symptoms, side effects, psychological changes, lifestyle, and medical decision-making. Comprehensive analysis of the psychometric properties should be performed and reported to assist clinicians and researchers in choosing the most appropriate instruments. We encourage routine inclusion of test-retest reliability, criterion validity, responsiveness, floor/ceiling effects, interpretability, time needed to complete, and reading level. We also recommend that authors provide full instrument information (list of all items, instructions for administration and scoring) for accurate clinical application. Comparative studies of different instruments in the same population can help identify the best or most appropriate instrument for a given context. Lastly, we want to see more cross-cultural research to broaden the application of self-efficacy in various populations and examine ethnic and socioeconomic variations.

## Conclusions

In this systematic review, we summarized and evaluated the psychometric parameters of 15 currently available instruments for assessing self-efficacy in cancer patients. The information reported here could be a resource for clinicians and researchers by helping them understand the strengths and limitations of the instruments and select the most appropriate tool for cancer care and innovation. Knowing the rigor and suitability of the instrument can also guide their efforts to determine the factors that influence a patient’s capacity for self-efficacy. Additional research is needed to strengthen the practicality and applicability of the instruments.

## Additional file

Additional file 1: **Table S1.** Psychometric properties of included instruments. **Table S2.** Quality criteria for psychometric properties of self-efficacy instruments for cancer patients. (DOCX 31 kb)

## Abbreviations

BCSES: Breast cancer self-efficacy scale
CASE-cancer: Communication and Attitudinal Self-Efficacy for Cancer
CFA: Confirmatory factor analysis
C-SUPPH: Strategies Used by People to Promote Health-Chinese version
CTT: Classical test theory
EFA: Exploratory factor analysis
IRT: Item response theory
OTSES-CA: Opioid-Taking Self-Efficacy Scale-Cancer
PSEFSM: Perceived Self-Efficacy for Fatigue Self-Management instrument
QOL: Quality of life
SESPRM-LC: Self-efficacy Scale for Rehabilitation Management for postoperative lung cancer patients
SICPA: The Standford Inventory of Cancer Patient Adjustment
SMSFS-A: Self-Efficacy in Managing Symptoms Scale-Fatigue Subscale for patients with advanced cancer
