# Supplementary material for: Psychometric properties and performance of existing self-efficacy instruments in cancer populations: a systematic review
Source: Health Qual Life Outcomes. 2018 Dec 27;16:241. doi: 10.1186/s12955-018-1066-9 (PMC6307141; doi:10.1186/s12955-018-1066-9)
Supplement: Supplementary file 1 — Table S1. Psychometric properties of included instruments. Table S2. Quality criteria for psychometric properties of self-efficacy instruments for cancer patients. (DOCX 31 kb) [file 12955_2018_1066_MOESM1_ESM.docx]

**Additional file 1**

**Table S1** Psychometric properties of included instruments

| Instrument | Reliability | | Validity | | | Interpretability |
| --- | --- | --- | --- | --- | --- | --- |
|  | Internal consistency | Test-retest | Content | Construct | Criteria |  |
| SICPA | *Cronbach's alpha*  of total scale:0.92;  of subscales:0.77~0.92 | One-week  of total scale:0.95;  of subscales: 0.70~0.97 | NR | NR | *Concurrent validity*  Affective management efficacy  and POMS distress scales  r:-0.61~-0.73;  activity management efficacy  and POMS vigor r=0.65;  POMS r=-0.73 | 183.33±35.8(one group pretest) |
| SUPPH | *Cronbach's alpha*  Of total scale:0.93~0.95  of subscale: 0.76~0.92 | 2 weeks  of total scale:0.94. | 47 cancer patients write the  items;  6 experts reviewed the items  and 100% agreement. | *EFA*  Four factor solution, (81% of total variance),  factors r=0.27~0.56.  Two factor solution, (81.3% of total variance),  factor loadings: 0.41~0.88.  *Convergent validity:*  HBS r=0.61,RGEI r=-0.38, FACT  r=0.36~0.59; BSI r=-0.29~-0.39, POMS  r=-0.27~-0.51, SDS r=-0.25~-0.32.  Generalizability coefficients=0.94~0.97 | NA | Effect size: 0.01~0.141. |
| SEAC | *Cronbach's alpha*  Of subscale: 0.73~0.81. | NA | 5 experts reviewed. | *EFA*  Three factor solution, (43.6% of total  variance), factor loadings: 0.42～0.90.  *CFA*  Three subscale of GFI:0.93~0.96; of  CFI:0.99~1.  *Divergent validity*  HADS depression r=-0.39~-0.41; anxiety  r=-0.32~-0.58. | NA | Of subscale:  76±2.2/63.3±2.3/78.4±1.7 |
| SESCI | *Cronbach's alpha*  Of total scale: 0.95 | NA | NR | Factors r =0.71～0.86. | NA | Of subscales:  56.1±24.3/  59.3±23.1/56.4±28.4 |
| CASE  -cancer | *Cronbach’ s alpha*  ≥0.76 | NA | 50 cancer patients in pilot  test assessed the scale | *EFA*  Three factor solution, (50.5% of total  variance), factor loadings:0.53~0.77.  *Divergent validity*  PPC r=0.22~0.60.  *DIF analysis*  All items performed equally among low literate and functionally literate patients. | NA | NR |
| OTSES-CA | *Cronbach's alpha*  Of total scale: 0.95;  of subscales:0.83~0.93;  Item-total correlations:  0.49~0.75. | 2-week  of subscales: 0.68~0.82 | 19 patients qualitative interview.  A panel of three native  Chinese-speaking experts reviews and achieved 94% agreement. | *EFA*  Four factor solution (73.16%), factor loadings:  0.87~0.43. | *Concurrent validity*  Adherence to an around-the-clock analgesic regimen r=0.22; BPI-SF pain intensity subscale r=0.25, pain relief subscale r=0.35. | 7.56±1.78 |
| CBI-B | *Cronbach's alpha*  Of total scale:0.84/0.88. | NA | Based on CBI | *EFA*  Four factor solution, factor loadings:  0.32~0.90.  *CFA*  χ^2^/df=5.37, RMSEA=0.07, SRMR=0.04,  CFI=0.95;  *Convergent validity*  FACT r=0.40~0.60;LOT-revised optimism  scale r=0.37~0.56; SWLS r =0.48;  COPE(problem-focused coping)r=0.18;  *Divergent validity*  SIP r=-0.38;COPE( avoidant coping) r=-0.32;  self-blame scale r=-0.33; RCS r=-0.28; Zung  depression scale r=-0.52; CES-D r=-0.55.  FACT-fatigue r=-0.39, anemia scale r=-0.45. | NA | 80.6±16.8(pre-intervention)/ 83.8±16.1(post-intervention) |
| PSEFSM | *Cronbach’ s alpha*  of total scale:0.92 | NA | 13 experts reviewed the scale | *Structural modeling* indicates a good model fit, explained 80% of the outcome variable and χ^2^=0.00, *P*=1.0, df=15,RMSEA=0.00. Partial mediation indicates that the severity of fatigue indirectly influences PFS by its effect on PSEFSM.  *Generalizability*  Measurement scores for the construct of PSEFSM were the same for lung cancer and other cancer population groups. | NA | 6.43±2.25 |
| SESSM-B | *Cronbach's alpha*  Of total scale: 0.78;  of subscales: 0.61~0.79;  item-total correlation:  0.28~0.57. | NA | 6 patients review  10 experts reviewed the  scale. | *EFA*  Five factor solution, (69.8% of total variance), factor loadings: 0.54～0.90. | *C**oncurrent validity*  EORTC QLQ-C30 r =0.09～0.3; EORTC BR23 r=0.2～0.28. | NR |
| BCSES | *Cronbach’ s alpha*  Of total scale:0.89;  inter-item correlations:  0.3~0.6;  *item-total correlations*:  0.49~0.75 | NA | 12 BCSs (at least two year  post-treatment) assessed the  scale.  5 experts reviewed the scale,  item-CVI=1 | *EFA*  One factor solution, (44% of total variance),  factor loadings: 0.52~0.78.  *Convergent validity*  IWB r=0.43, CES-D r=-0.4,AFI r=0.42,  CARS r=-0.4, communication with HCP  r=0.33, ENRICH-MSS r=0.31, FACT-fatigue  r=0.35, Sexual index scale r=0.26, STAI  r=-0.49～-0.52, Symptom bother scale  r=-0.26. | *Predictive validity*  Well-being(R^2^=47%) | NR |
| C-SUPPH | *Cronbach's alpha*  Of total scale =0.98;  of subscale: 0.83~0.97 | NA | 14 clinical nurses and 5  patients reviewed the items. | *CFA*  Factors r=0.77~0.9. CFI=0.94, TLI=0.94,  RMSEA=0.05, SRMR=0.04.  *Measurement invariance*  The factorial structure and factor loadings  (relationships between items and their  underlying factors) of C-SUPPH were  invariant across difference gender and age  groups as well as education and monthly  income levels. | NA | 84.11±24.47 |
| EBSES | *Cronbach's alpha*  Of total scale =0.93;  of subscales: 0.83~0.93; *Item-total correlations：*  :0.49~0.75.. | 2-week  of total scale: 0.67  of all items:0.44~0.65. | Expert consultation | *EFA*  Two factor solution (76.6%), factor loadings:  0.60~~0.97.  *Discriminant validity*  FACT-B+ 4 r=0.31; DASH r=-0.34 | General barriers scale r=0.61. | NR |
| SMSES-BC | *Cronbach's alpha*  of total scale =0.96;  of subscales: 0.88~0.95;  *Item-total correlations:*  0.56~0.81. | 2-week  of total scale: 0.73  of all items:0.40~0.78. | 6 experts reviewed,  agreement ranged from 0.83-1.00 | *EFA*  Five factor solution (70.2%),factor loadings: 0.45~0.79  *CFA*  Factor loading: 0.58~0.84. | GSES r=0.37~0.4 | NR |
| SMSFS-A | NA | 1-week  of total scale:acceptable agreement(mean difference=-0.75) | 5 experts reviewed, CVI=1  10 patients reviewed the  items | NA | NA | NR |
| SESPRM-LC | *Cronbach's alpha*  of total scale =0.86;  of subscales: 0.70~0.89;  *Mean inter-Item correlations:*  of total scale =0.29.  *Marginal reliability*  of total scale =0.93  of subscales:0.57~0.93 | 2-week  of total scale: 0.89  of all items:0.65-0.89 | 16 experts reviewed, S-CVI=0.88  10 patients reviewed the items | *EFA*  Six factor solution, (60.8% of total variance), factor loadings: 0.46～0.88.  *CFA*  CFI=0.90, TLI=0.88,RMSEA=0.06  IRT  βik : -7.37～1.07  92.6% of all items have very high discrimination (α>1) | GSES r=0.47  *Predictive validity*  FACT-L total r=0.24 | Effect size: 1.08 |

Abbreviations: AFI, Attentional Function Index; BCSs: Breast Cancer Survivors; BSI, Brief Symptom Inventory; BPI-SF, Brief Pain Inventory Short Form; CARS, Concerns About Recurrence Scale; CES-D, Center for Epidemiologic Studies Depression Scale; CFA, Confirmatory Factor Analysis; CFI, Comparative Fit Index; CVI, Content Validity Index; DASH, Disabilities of the Arm, Shoulder and Hand; DIF, Differential Item Functioning; ECOG, Eastern Cooperative Oncology Group; EFA, Exploratory Factor Analysis; ENRICH-MSS, ENRICH Marital Satisfaction Scale; EORTC QLQ-BR23, European Organization of Research and Treatment of Cancer quality of life questionnaire for Breast cancer specific module; EORTC QLQ-C30, European Organization of Research and Treatment for Cancer Quality of Life Questionnaire Core; FACT, Functional As­sessment of Cancer Therapy; GFI, Goodness of Fit Index; HADS, Hospital Anxiety and Depression scale; HBS, Health Behavior Scale; HCP, HealthCare Provider; IWB, Index of Well-Being; LOT, Life Orientation (Optimism) Test; NRS, Numerical Rating Scale; PFS: Physical Functional Status; POMS, Profile of Mood States; PPC: Perceived Personal Control scale; RCS, Religious Coping Scale; RGEI, Revised Grief Experience Inventory; RMSEA, Root-Mean-Square Error of Approximation; SDS: Symptom Distress Scale ;SIP: Sickness Impact Profile; SRMR, Standardized Root-Mean-square Residual; STAI, State-Trait Anxiety Inventory; SWLS, Satisfaction with Life Scale; TLI: the Tucker-Lewis index.

**Table S2** Quality criteria for psychometric properties of self-efficacy instruments for cancer patients

| Property | Sub-property | Definition | Quality criteria | |
| --- | --- | --- | --- | --- |
|  |  |  | Positive rating(＋) | Negative rating(－) |
| Reliability | Internal consistency | The extent to which items in a (sub) scale are inter-correlated, thus measuring the same construct.  The degree to which items of a tool measure the same construct (e.g., homogeneity of items); assessed by Cronbach’s alpha and item-to-total correlations | Cronbach’s alpha for each dimension or the total scale was between 0.70~0.95; or item-to-total correlations≥0.30. | Cronbach’s alpha has a value > 0.95 (indicates redundancy) or < 0.70 (indicates lack of correlation); or item-to-total correlations＜0.30. |
|  | Test-retest(Reproducibility) | The short-term stability of a tool over time; assessed by administering the instrument to respondents on 2 different occasions. | Pearson’s r, ICC or Kappa coefficients was ≥ 0.7 and an explicit time interval of repeated measures was provided. | Pearson’s r, ICC, or Kappa was < 0.70, or the explicit time interval of repeated measures was not reported |
| Validity | Content | The extent to which the content of a tool is relevant and representative of the targeted conceptual domain it is intended to cover for a particular assessment purpose | A clear description is provided of qualitative evidence from interviews with patients and clinicians, pretesting with patients, expert opinion and literature review; patients and clinicians involved in the development stage and item generation; or CVI ≥0.7 | A clear description of these aspects was lacking; or CVI ＜0.7 |
|  | Construct | The extent to which scores on a particular questionnaire related to other measures in a manner that is consistent with theoretically derived hypotheses concerning the concepts that are being measured. | A clear description is provided of the process and results of factor analysis (EFA or CFA) or the degree to which a tool is correlated with other measures of similar or dissimilar constructs; and factor analyses performed on adequate sample size(≥100). | This information was inadequate or missing; and factor analyses performed on inadequate sample size(＜100). |
|  | Criterion | The extent to which the scores of the instrument correlate with a gold standard or another self-efficacy instrument, including concurrent validity and predictive validity. | The correlation coefficient with related instrument was ≥ 0.7(P≤0.05). | The correlation coefficient with related instrument was ＜0.7 or non-significant correlations (P＞0.05). |
| Interpretability | | The degree to which one can assign qualitative meaning or quantitative scores. | Mean scores and SD of at least one group or cut-offs and reference scores of the instrument were provided. | This information was inadequate or missing. |
| Floor/Ceiling effects | | The number of respondents who achieved the lowest or highest possible score. | Proportion of sample with the highest or lowest scores ≤15% | Proportion of sample with the highest or lowest scores ＞ 15% |
| Responsiveness | | The ability of a tool to detect clinically significant change over time, in correlation with other measures, or before and after an intervention of known efficacy. Calculation of effect size should be included. | Presence | Absence |

ICC=intra-class coefficients; CVI= content validity index; EFA=Exploratory factor analysis; CFA=Confirmatory factor analysis; NR= not reported; SD=standard deviations
